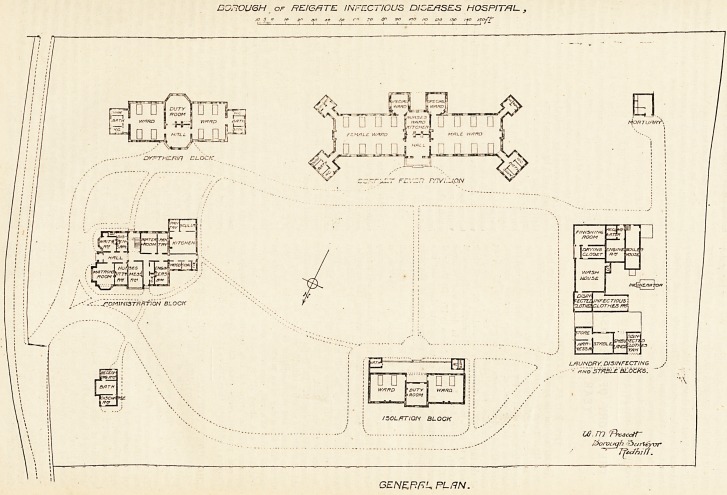# Hospital Construction

**Published:** 1900-04-21

**Authors:** 


### The Institutional Workshop.

### HOSPITAL CONSTRUCTION.

### THE INFECTIOUS DISEASES HOSPITAL, REIGATE.

Including the mortuary and the discharging-rooms this hospital consists of seven blocks, and these are arranged in the form of an irregular parallelogram. The blocks are sufficiently far apart from each other, and there is a large open space in the centre. Practically it may be said that the wards lie east and west, and so have a northern and southern exposure in their long diameters.

The scarlet-fever pavilion is divided in its centre by a hall and by the nurses' ward kitchen. On one hand is the men's ward, and on the other the women's. Each ward contains eight beds. Each bed is provided with 156 ft. of floor space, and a little over 2,000 cubic feet of air space. From centre to centre of the beds the distance is 12 ft. A single-bedded room projects southwards, and has been cleverly arranged so that cross-ventilation can be obtained. We should have liked to see a window in the southern exposure also of this small room. The closets and bath-rooms are correctly placed and correctly designed. Altogether this block is extremely good.

East of the scarlet-fever block is that for diphtheria. This contains four beds for each sex, and, as in the block already described, the wards are separated by a hall and by a nurses' duty-room. There is efficient cross ventilation in the ward itself.

North of the diphtheria ward is the administration block. It is carefully planned, containing all essential offices and rooms for the staff.

The isolation block contains a nurses' room and two wards, each ward having two beds. The block is intended for cases of typhoid fever, or for such cases as find their way into all fever hospitals—namely, those whose disease is uncertain on admission. A verandah runs along the whole length of the southern aspect of this block, and at one end is a bath-room and at the other a closet. These are too much in evidence from the ward windows; and if placed near this position at all the bath should have been placed about ten feet further east and the closet about the same distance further west. The chimneys ought to have been in the ends of the wards, and a window should have taken the position of the present chimneys.

The laundry, stable, and ambulance house occupy the extreme west side of the parallelogram. The component parts are well arranged. The drying-room, however, seems small for its purpose. The space between the doors and the wall next the engine-room can hardly exceed 12 ft., so that the drying horses cannot be more than 6 ft. long. There is a disinfecting-room, and also an incinerator for the destruction of sweepings and typhoid excreta.

The discharging-room is very good. Patients about to leave the hospital will undress in the receiving-room, pass into the bath-room, and from it into the discharging-room.

The ventilation of the various wards has been well attended to. Each window is provided with a fanlight, and also double hung sashes. Special fresh air inlets are placed on the floor level. The walls are covered with Portland and Parian cement, so that cleaning can be done easily and efficiently.

The system of drainage is a good one. Each block is separately dealt with, and the main drain from each is conducted separately to the sewer. Each main drain has an intercepting chamber and an automatic flushing tank. The water supply is from the East Surrey Water Company's main, and the pressure is sufficient to cope with a fire should there ever be an outbreak. The lighting is by electricity generated in the hospital. Taken as a whole the hospital may be pronounced a good one, and Reigate is fortunate in possessing it. The architect is Mr. W. H. Prescott, of Redhill. The cost of the building is not given.

### STRUCTURAL IMPROVEMENTS AT QUEEN CHARLOTTE'S HOSPITAL.

Queen Charlotte's Hospital has been greatly improved by the structural additions and adaptations that have been in progress for some time past. A new storey provides accommodation for the sisters forming the permanent nursing staff. Each has the comfort and pleasure afforded by a spacious, light, and well-furnished private apartment. Bath-room, lavatories, &c., for their use are placed on the same floor, but apart from the main corridor. Access is given to this storey by a spiral stone staircase, and the whole is shut off from that part of the building occupied by the patients.

Three storeys are now at the disposal of the authorities for patients' use. The whole of the top floor is divided into different sized wards, with their complement of kitchen, larder, bath, lavatories, linen, and sisters' rooms, &c. But a complete and self-contained suite of labour-rooms has been arranged on both the floors beneath, which are shut off entirely from the ordinary wards. Patients are taken into a large warmed bath-room, and after a toilet pass on to a comfortable and cheery waiting-room, provided with every comfort, and presided over by a trained nurse. Adjoining are a couple of large labour-rooms, each containing two beds. The new passenger lift has added greatly to the comfort of all concerned. A sitting-room, bedroom, and bath-room, arranged *en suite*, have been allotted to the matron on the ground floor. Close by a nurses' sitting and lecture room has been prepared. The one is separated from the other by curtains. The lecture-room is constantly used, the short period spent by the candidate in training necessitating the most careful husbanding of every hour.

Much has been done in the basement. The soiled linen-room has been considerably enlarged. The clean linen is sorted and baked in the new disinfector before being put away to the cupboards. Mattresses and bedclothes are taken to another room, baked, and passed through the disinfector out into this one between each case. The kitchens, larders, pantries, and store-rooms have been renovated; a good effect has been obtained by the use of "Newellite," a wall decoration something like opaline in appearance. Gauze windows and door

INFECTIOUS DISEASES HOSPITAL, REIGATE.—(*See opposite page.*)

have been added in places where it is desirable to secure better ventilation, and the pipes of the new hot-water supply have been encased in asbestos to prevent their over-heating the corridor through which they pass.

Throughout the hospital the most modern sanitary fixtures have been placed. The walls are of painted cement, and the floors polished wood. "Newellite" and terrazzo have been freely used in bath-rooms, passages, &c., whilst incandescent gas furnished a very good light all over the building.

## Figures and Tables

**Figure f1:**